# PlGF mediates neutrophil elastase-induced airway epithelial cell apoptosis and emphysema

**DOI:** 10.1186/s12931-014-0106-1

**Published:** 2014-09-05

**Authors:** Hsin-Han Hou, Shih-Lung Cheng, Kuei-Pin Chung, Shu-Chen Wei, Po-Nien Tsao, Hsuan-Hsuan Lu, Hao-Chien Wang, Chong-Jen Yu

**Affiliations:** Departments of Internal Medicine, National Taiwan University Hospital, No. 7, Chung-Shan South Road, Taipei, Taiwan; Department of Internal Medicine, National Taiwan University, College of Medicine, Taipei, Taiwan; Department of Internal Medicine, Far Eastern Memorial Hospital, New Taipei City, Taiwan; Department of Chemical Engineering and Materials Science, Yuan-Ze University, Taoyuan, Taiwan; Departments of Laboratory Medicine, National Taiwan University Hospital, No. 7, Chung-Shan South Road, Taipei, Taiwan; Departments of Pediatrics, National Taiwan University Hospital, No. 7, Chung-Shan South Road, Taipei, Taiwan

**Keywords:** Placenta growth factor, Chronic pulmonary obstructive disease, Neutrophil elastase, Apoptosis, Emphysema

## Abstract

**Background:**

Chronic pulmonary obstructive disease (COPD) has become the fourth leading cause of death worldwide. Cigarette smoking induces neutrophil elastase (NE) and contributes to COPD, but the detailed mechanisms involved are not fully established. In an animal model of pulmonary emphysema, there are increased expressions of placenta growth factor (PlGF) and lung epithelial (LE) cell apoptosis. This study hypothesized that excessive NE may up-regulate PlGF and that PlGF-induced LE apoptosis mediates the pathogenesis of pulmonary emphysema.

**Methods:**

Human bronchial epithelial cells, BEAS-2B, and primary mouse type II alveolar epithelial cells were treated with NE. The PlGF promoter activity was examined by luciferase activity assay, while PlGF expression and secretion were evaluated by RT-PCR, Western blotting, and ELISA. Both cell lines were treated with PlGF to evaluate its effects and the downstream signaling pathways leading to LE cell apoptosis. PlGF knockout and wild-type mice were instilled with NE to determine the roles of PlGF and its downstream molecules in NE-promoted mice pulmonary apoptosis and emphysema phenotype.

**Results:**

The transcriptional factor, early growth response gene-1, was involved in the NE-promoted PlGF promoter activity, and the expression and secretion of PlGF mRNA and protein in LE cells. PlGF-induced LE cell apoptosis and NE-induced mice pulmonary apoptosis and emphysema were mediated by the downstream c-Jun N-terminal kinase (JNK) and protein kinase C (PKC)δ signaling pathways.

**Conclusion:**

The NE-PlGF-JNK/PKCδ pathway contributes to the pathogenesis of LE cell apoptosis and emphysema. PlGF and its downstream signaling molecules may be potential therapeutic targets for COPD.

**Electronic supplementary material:**

The online version of this article (doi:10.1186/s12931-014-0106-1) contains supplementary material, which is available to authorized users.

## Background

Chronic pulmonary obstructive disease (COPD) is predicted to become the fourth leading cause of death worldwide by 2030 [1,2]. Due to the aging population and increasing number of smokers, the burden of medical and social resources for COPD is estimated to be US$47 trillion by 2030 [3]. Although there are many mediators (i.e., inflammatory cells, lipids, reactivate oxygen species, nitric oxide, peptides, chemokines, cytokines, growth factors, and proteases) and cellular pathways (e.g., inflammation, apoptosis, senescence and repair) involved in the pathogenesis of COPD, increasing evidence indicates that proteases provide vital contributions to all mediators and cellular pathways [4,5]. However, to date, the detailed pathogenic mechanisms of protease-mediated COPD are not fully understood [3,6].

In developed countries, the major factor for the pathogenesis and progression of COPD is cigarette smoke (CS). Exposure to CS results in chronic inflammation, elevated oxidative stress, and protease-anti-protease imbalance within the respiratory system [7]. The protease-anti-protease imbalance is triggered by the infiltration of inflammatory cells like neutrophils, macrophages, and CD8^+^ T lymphocytes [7-11]. Proteolytic enzymes of neutrophils and macrophages, neutrophil elastase (NE), and matrix metalloproteinase (MMP)-12, degrade their respective inhibitors. Thus, the interaction promotes protease-anti-protease imbalance and destroys the pulmonary parenchyma with alveolar space dilatation, i.e. emphysema, which is a major component of COPD [12].

Neutrophil elastase is a secreted serine protease that degrades extracellular matrix like elastin, which contributes to the recoil capacity of alveoli [13]. Other than proteolytic activity, NE up-regulates elafin, interleukin-8, MUC4, and MUC5AC, and promotes the secretion of mucin in LE cells [14-18]. Excessive NE also results in LE cell apoptosis through protease-activated receptor (PAR)-1, which is abrogated by treatment with retinoic acid [19,20].

Apoptosis of LE cells results in the loss of lung parenchyma and is a potential pathogenic mechanism for emphysema and COPD [21]. Placenta growth factor (PlGF) induces apoptosis of type II alveolar epithelial cells (AEC II) such that PlGF transgenic mice develop a phenotype of pulmonary emphysema [22]. PlGF is a member of the vascular endothelial growth factor family that promotes angiogenesis [23,24]. PlGF expression is abundant in the placenta, heart, lungs, thyroid, brain, and skeleton muscle during fetal development, but declines in adulthood [25]. Higher levels of PlGF have been shown in serum and broncho-alveolar lavage (BAL) fluid of COPD patients and the PlGF levels is inversely proportional to lung function deterioration [26]. Porcine pancreatic elastase (PPE), a recombinant porcine elastase for the animal model of emphysema, has also been shown to increase PlGF expression in LE cells and promote LE cells apoptosis [27]. However, the role of NE in human COPD has not been established.

Under the hypothesis that NE, like PPE, up-regulates PlGF expression and leads to LE cell apoptosis and pulmonary emphysema. This study demonstrates that the NE-promoted PlGF expression and secretion in LE cells and lungs. Early growth response gene (Egr)-1 is a transcriptional factor responsible for the up-regulation of PlGF by NE in LE cells. PlGF induces apoptosis through the c-Jun N-terminal kinase (JNK) and protein kinase C (PKC)δ signaling pathways. Ablation of PlGF protects mice from NE-induced pulmonary apoptosis and emphysema. Thus, NE-induced PlGF and the downstream JNK/PKCδ signaling pathways contribute to the pathogenesis of pulmonary emphysema and COPD. Both PlGF and its downstream signaling pathways may be potential therapeutic targets for COPD.

## Materials and methods

### Reagents

Rabbit antibodies for phosphor-P38 MAPK (p-P38 MAPK), P38 MAPK, MTF-1, p-JNK and p-PKCδ were obtained from Cell Signaling Technology (Beverly, MA, USA). Antibodies for PlGF, JNK, PKC, and Egr-1, mouse and human PlGF siRNA, mouse and human PKCδ siRNA, and the corresponding scramble siRNA were purchased from Santa Cruz (Santa Cruz, CA, USA), while NE was purchased from Abcam (Cambridge, MA, USA). Trizol reagent, SuperScript III Reverse Transcriptase and Lipofectamine 2000 were obtained from Invitrogen (Carlsbad, CA, USA). Mouse antibody for beta-actin and rabbit antibody for HIF-1alpha were purchased from Genetex (Irvine, CA, USA). Human and mouse recombinant PlGF protein and an enzyme-linked immuno-sorbent assay (ELISA) kit were obtained from R and D Systems (Minneapolis, MN, USA).

A dual-luciferase reporter assay system was obtained from Promega (Madison, WI, USA). Hematoxylin and Eosin, Chromatin immuno-precipitation (ChIP) Assay Kit, and EZ-Zyme Chromatin Prep Kit were purchased from Merck-Millipore (Boston, MA, USA). An *in situ* cell Death Detection Kit and X-tremeGENE HP DNA Transfection Reagent were purchased from Roche (Mannheim, Germany). The FITC Annexin V apoptosis detection Kit I was obtained from BD Biosciences (San Jose, CA, USA). The JNK inhibitor, SP600125, was obtained from Enzo Life Science (Plymouth Meeting, PA, USA). A SuperSensitive Polymer-HRP IHC Detection System was purchased from Biogenex (Fremont, CA, USA).

### Animals

This study conformed to the Guidelines for the Care and Use of Laboratory Animals published by the United States National Institutes of Health (NIH Publication No. 85–23, revised 1996). All of the animal experiments were approved by the Institutional Animal Care and Use Committee (IACUC) of the Laboratory Animal Center, College of Medicine and Public Health of National Taiwan University. Eight-week-old male C57BL/6 wild type (WT) mice were purchased from the Laboratory Animal Center, College of Medicine and College of Public Health, National Taiwan University. The PlGF knockout (KO) mice in B6 background were provided by Dr. Po-Nien Tsao (National Taiwan University, Taiwan).

### Cell culture

Human bronchial epithelial cells, BEAS-2B (ATCC number CRL-9609), were cultured in F12 nutrient mixture (Carlsbad, CA, USA) with 0.5 ng/ml recombinant epidermal growth factor, 500 ng/ml hydrocortisone, 0.005 mg/ml insulin, 0.035 mg/ml bovine pituitary extract, 500 nM ethanolamine, 500 nM phosphoethanolamine, 0.01 mg/ml transferrin, 6.5 ng/ml 3, 3′, 5-triiodothyronine, 500 ng/ml epinephrine, 0.1 ng/ml retinoic acid, 10% FCS 100 unit/ml penicillin, and 100 μg/ml streptomycin in a humidified 95% air-5% CO_2_ incubator at 37°C. Mouse primary type II alveolar epithelial cells (AEC II) and culture medium were purchased from chi scientific (Maynard, MA, USA). Primary normal human bronchial epithelial (NHBE) cells were kindly provided by Dr. Reen Wu at University of California, Davis.

### Plasmids

Human genomic DNA was extracted from BEAS-2B by a Quick-gDNA MiniPrep kit (Zymo Research, CA, USA). The 2.0 kb human PlGF promoter region was amplified from human genomic DNA using polymerase chain reaction (PCR) performed with Hi Fi *Taq* DNA polymerase (Geneaid, Taipei, Taiwan) as follows: 2 minutes at 94°C, then 15 sec at 94°C, 30 sec at 59°C, and 2 min and 30 sec at 72°C for 35 cycles. The primers for 2.0 kb human PlGF promoter region were 5′-GC*G GTAC C*CA AAC TCA TAC ACA ATA GAC-3′ (forward primer; italic, *Kpn*I site) and 5′-TT*A AGCT T*CC GTA GGT AAG GCT GTG GCT-3′ (reverse primer; italic, *Hind*III site). The amplified DNA fragments were cloned into pGL3 vector (Promega, WI, USA) and the sequences were confirmed by DNA sequence analysis. The pGL3 with mouse PlGF promoter was as previously described [27].

### Enzyme-linked immuno-sorbent assay (ELISA)

Cellular medium from BEAS-2B and AEC II, and BAL fluid from mice were analyzed by a PlGF ELISA kit (R & D, MN, USA) according to the manufacturer’s instructions.

### Luciferase reporter assay

The BEAS-2B and AEC II were co-transfected with the pGL3-PlGF promoter and pRenilla for 24 h via Lipofectamine 2000 and X-tremeGENE HP DNA Transfection Reagent, and then collected and analyzed on a dual-luciferase reporter assay system (Promega, WI, USA) using a lumicounter Packard BL10000 according to the manufacturer’s instructions.

### Protein extraction and immuno-blot analysis

The BEAS-2B and AEC II were lysed using RIPA lysis buffer (Genestar, Taipei, Taiwan), containing 1% NP-40, 0.1% SDS, 150 mM sodium chloride, 0.5% sodium deoxycholate, and 50 mM Tris with a protease inhibitor cocktail (Bionovas, Toronto, Canada) and PhosSTOP (Roche, Basilea, Switzerland). The cell lysates were centrifuged at 12,000 rpm for 5 min and the resulting supernatant was collected.

The extracted protein was quantified by protein assay. Equal amounts of protein were separated using 10% SDS-polyacrylamide gel electrophoresis and transferred to Immobilon-P membranes (Millipore, MA, USA). After blocking with 5% skimmed milk, the membranes were incubated with various primary antibodies and then incubated with the corresponding secondary antibodies. The protein bands were detected using an Immobilon Western Chemi-luminescent HRP Substrate (Millipore, MA, USA) and quantified by the ImageQuant 5.2 software (Healthcare Bio-Sciences, PA, USA).

### Terminal deoxynucleotidyl transferase dUTP nick end labeling (TUNEL) assay

The BEAS-2B and AEC II, and OCT-embedded lung tissue from the mice were analyzed for the apoptosis level using an *in situ* cell Death Detection Kit (Roche, Basilea, Switzerland) according to the manufacturer’s instructions. Fluorescence-positive cells were photographed by a Leica DM 4000B microscope (Leica, Solms, Germany).

### Flow cytometry analysis

The BEAS-2B and AEC II were analyzed on a FITC Annexin V apoptosis detection Kit I (Franklin Lakes, NJ, USA) according to the manufacturer’s instructions. The FITC-positive cells were analyzed using a FACS Calibur flow cytometer (Becton Drive, NJ, USA).

### Immuno-histochemistry (IHC) assay

Paraffin was removed from paraffin-embedded tissue sections by xylene, dehydrated by ethanol, and re-hydrated by PBS. After treatment with 3% H_2_O_2_, the sections were applied to a SuperSensitive Polymer-HRP IHC Detection System (Biogenex, CA, USA) and incubated with PlGF, p-JNK, and p-PKCδ antibodies as primary antibodies. The stained-sections were photographed using a Leica DM 4000B microscope (Leica, Solms, Germany).

### Hematoxylin and eosin (H and E) staining

Paraffin was removed from paraffin-embedded tissue sections by xylene, dehydrated by ethanol, and re-hydrated by PBS. Sections stained with H and E were photographed by a Leica DM 4,000 B microscope (Leica, Solms, German).

### NE-induced emphysema

The dose of NE was four-fold higher than that of porcine pancreatic elastase according to previous report [28] and the methodology of intra-tracheal instilling NE was performed as previously described [29]. Briefly, eight-week-old mice were intra-tracheally given saline (CON), 400 mU/ml NE (NE), 400 mU/ml NE with 50 mg/kg JNK inhibitor SP600125 (NE SP), 3 mg/kg scramble siRNA (NE Si-Sc), 3 mg/kg mouse PKCδ siRNA (NE Si-PK) and 3 mg/kg PlGF siRNA (NE Si-Pl) weekly for one month. The dose of siRNA instillation was according to a previous study [27,30]. Each experimental group had five mice and the processing of lung tissues and BAL fluid were performed as previously described [27,29].

### Reverse-transcriptional (RT)-PCR assay

Total RNA of BEAS-2B and AEC II were extracted by Trizol Reagent (Invitrogen, CA, USA) according to the manufacturer’s instructions. Total RNA (5 μg) was used in the RT reactions using a SuperScript III Reverse Transcriptase kit (Invitrogen, CA, USA) according to the manufacturer’s instructions to synthesize the cDNA. The human PlGF and glyceraldehyde 3-phosphate dehydrogenase (GAPDH) cDNA fragments were amplified from the cDNA by PCR, performed with Dream *Taq* DNA polymerase (Fermentas, MA, USA) as follows: 5 min at 95°C, then 30 sec at 98°C, 30 sec at 59°C, and 1 min at 72°C for 35 cycles. The primers for 164 bp human PlGF cDNA fragment were 5′-GGC GAT GAG AAT CTG CAC TGT-3′ and 5′-GAA GAT GAA GCC GGA AAG GTG-3′. The primers for 530 bp human GAPDH cDNA fragment were 5′-GGG CGC CTG GTC ACC AGG GCT G-3′ and 5′-GGG GCC ATC CAC AGT CTT CTG-3′. The primer sets for mouse PlGF and GAPDH was as previously described [27].

### Chromatin immuno-precipitation (ChIP)

Genomic DNA fragment from BEAS-2B were prepared by the EZ-Zyme Chromatin Prep Kit (Millipore, MA, USA) and analyzed using the Chromatin immuno-precipitation (ChIP) Assay Kit (Millipore, MA, USA) to evaluate the associated levels of Egr-1 and PlGF promoter regions. The antibody of Egr-1 was used for immuno-precipitation and the primer set (5′-CAC TTT CCA AGA ATG CCT ATG TCC ATT C-3′ and 5′-TTA AGC TTC CGT AGG TAA GGC TGT GGC T-3′) were used to amplify the human PlGF promoter fragment according to the manufacturer’s instructions.

### Statistical analysis

The results were presented as mean ± SEM from five independent experiments and animals. The Mann–Whitney test was used to compare two independent groups. Kruskal-Wallis with Bonferroni post hoc analysis was used for multiple testing. Statistical analyses were performed using the SPSS version 8.0 (SPSS Inc., IL, USA). Statistical significance was set at *p* < 0.05.

## Results

### NE increased PlGF promoter activity by Egr-1 in LE Cells

**Figure 1:**
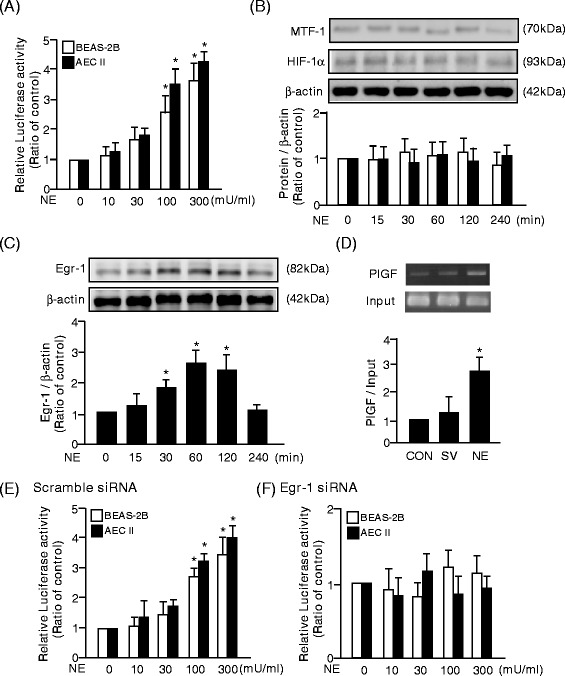
**NE-induced PlGF promoter activity is mediated by Egr-1. (A, E, and F)** The placenta growth factor (PlGF) promoter activity was evaluated by luciferase activity. **(B)** Hypoxia inducible factor (HIF)-1alpha and metal-regulatory transcription factor (MTF)-1, **(C)** Egr-1, and β-actin were analyzed by Western blot analysis. **(D)** The association of Egr-1 and PlGF promoter fragment was evaluated by chromatin immuno-precipitation assay. Data are presented as mean ± SEM. ^*^ *p* < 0.05 vs. vehicle-treated group.

The results revealed that treatment with 100–300 mU/ml NE for 24 h significantly increased PlGF promoter activity dose-dependently in human bronchial epithelial cells, BEAS-2B, and primary mouse type II alveolar epithelial cell (AEC II) (Figure 1A). Previous studies indicated that several conserved metal response elements (MRE) and hypoxia response elements (HRE) reside in mouse or human PlGF promoter regions [31,32]. However, treatment with 300 mU/ml NE did not alter the expression of mental-regulatory transcription factor (MTF)-1 and hypoxia inducible factor (HIF)-1α (Figure 1B).

There was a conserved Egr-1 response element in the human and mouse PlGF promoter regions near the transcriptional start site [32,33]. Western blotting revealed that 300 mU/ml NE challenge transiently increased Egr-1 expression in BEAS-2B (Figure 1C). By ChIP, treatment of 300 mU/ml NE for 1 h triggered the binding of Egr-1 and PlGF promoter fragments in BEAS-2B (Figure 1D) and pre-treatment with Egr-1 siRNA inhibited the NE-increased PlGF promoter activity in BEAS-2B and AEC II (Figures 1E and F). Thus, NE increased PlGF promoter activity through the association of Egr-1 and the PlGF promoter fragment.

### NE increased PlGF expression in LE Cells

**Figure 2:**
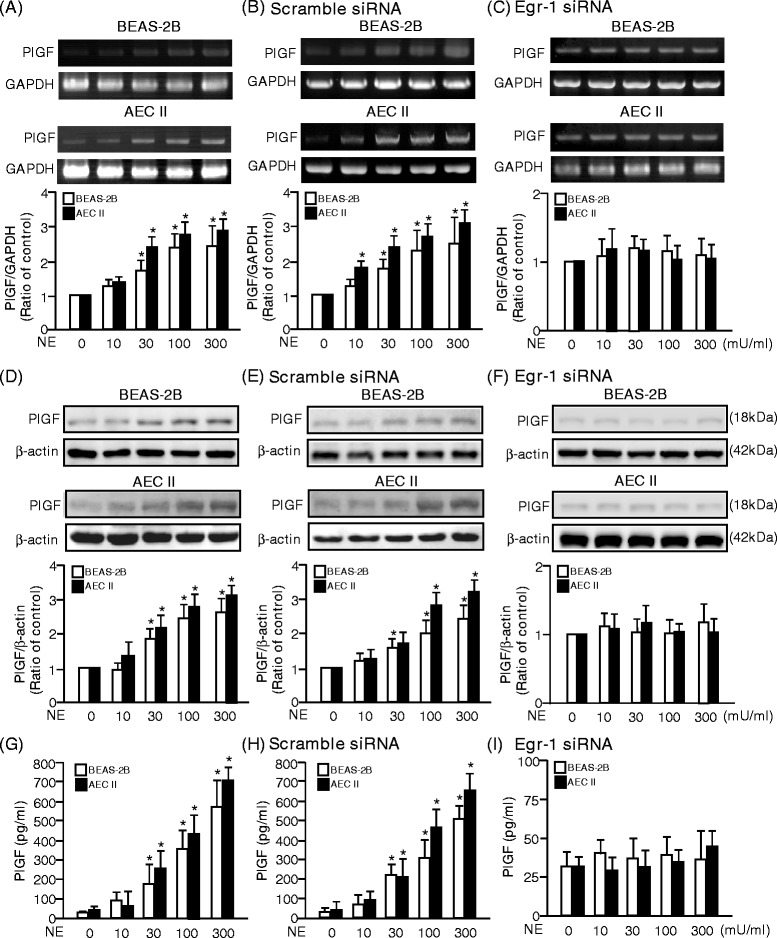
**NE-induced PlGF expression and secretion are mediated by Egr-1. (A-C)** The mRNA level of PlGF was determined by reverse-transcriptional polymerase chain reaction (RT-PCR) with primer sets for PlGF and GAPDH cDNA. **(D-F)** Cellular lysates were also subjected to Western blot analysis with antibodies for PlGF and β-actin. **(G-I)** The PlGF in the culture medium was detected by enzyme-linked immuno-sorbent assay (ELISA). Data are presented as mean ± SEM. ^*^ *p* < 0.05 vs. vehicle-treated group.

NE (100 mU/ml) had been reported to up-regulate elafin expression in A549 cells [14] and PlGF was majorly secreted by AEC II [22,34]. To test whether NE could induce PlGF expression, BEAS-2B and AECII were treated with of 0–300 mU/ml NE for 24 h. PlGF mRNA and protein level were increased after NE challenge in a dose-dependent manner and Egr-1 siRNA pre-treatment abrogated the NE-induced PlGF mRNA (Figure 2A-C) and protein (Figure 2D-F) expressions in BEAS-2B and AEC II. Moreover, Egr-1 siRNA also blocked the NE-induced PlGF secretion in medium of BEAS-2B and AEC II (Figure 2G-I).

Moreover, NE increased the PlGF expression in endothelial cell but not in fibroblast cell (Additional file 1 and Additional file 2: Figures S1A and S1B). Taken together, other than natural activity of proteolysis, NE increased the PlGF expressions and promoted PlGF secretion.

### PlGF induced apoptosis in LE Cells via JNK and PKCδ signaling pathways

A previous study indicated that 100 ng/ml PlGF induced MLE-15 cell apoptosis with an unknown mechanism [22]. It has been previously demonstrated that PlGF increased apoptosis in MLE-15 cells and BEAS-2B via JNK and p38 mitogen-activated protein kinase (MAPK) signaling pathways [27,35]. In order to confirm and explore the mechanisms underlying PlGF-induced LE cells apoptosis, BEAS-2B and AEC II were treated with 100 ng/ml recombinant PlGF for 24 h.

**Figure 3:**
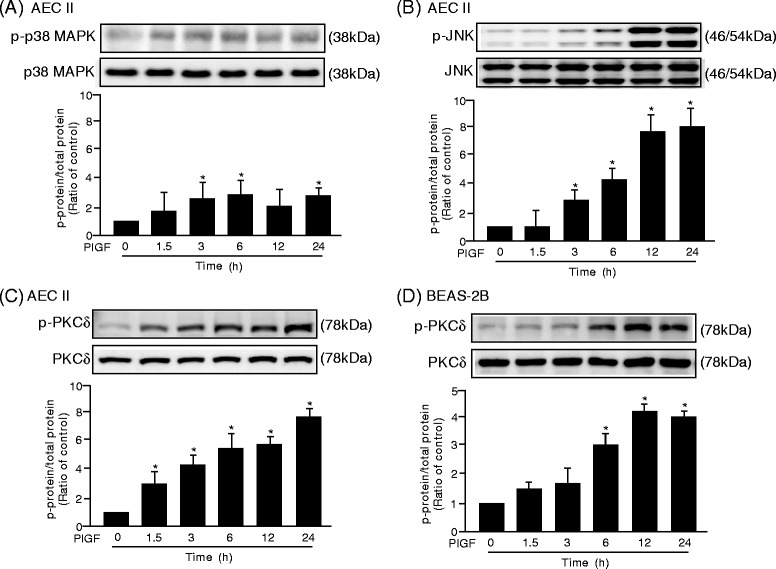
**PlGF activates JNK and PKCδ signaling pathways in LE cells. (A-C)** AEC II and **(D)** BEAS-2B were treated with 100 ng/ml recombinant human mouse PlGF respectively for 0–24 h. Cellular lysates were subjected to Western blot analysis with antibodies for phosphorylated p38 MAPK (p-p38 MAPK) and p38 MAPK **(A)**, phosphorylated JNK (p-JNK) and JNK **(B)**, phosphorylated PKCδ (p-PKCδ) and PKCδ **(C and D)**. Data are presented as mean ± SEM. ^*^ *p* < 0.05 vs. vehicle-treated group.

Although the results of Western blot analysis revealed that PlGF didn’t activate p38 MAPK significantly, PlGF induced a prolonged and enhanced phosphorylation of JNK and PKCδ in AEC II (Figure 3A-C). PlGF also activated PKCδ pathways in BEAS-2B (Figure 3D). Blockade of JNK or PKCδ signaling by JNK inhibitor, SP600125, or transfection with PKCδ siRNA had no effect on PlGF-activated PKCδ or JNK (Additional file 3: Figure S2), suggesting no crosstalk between PlGF-activated JNK and PKCδ signaling pathways.

**Figure 4:**
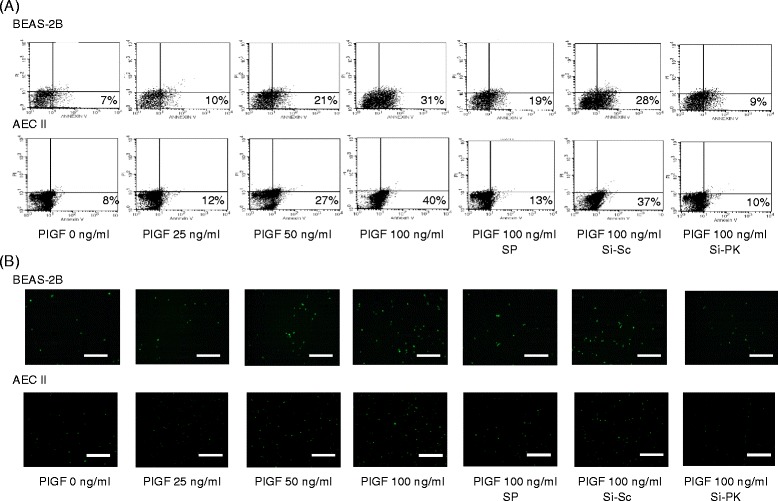
**PlGF triggers LE cell apoptosis via JNK and PKCδ signaling pathways.** The PlGF-induced apoptosis was evaluated by **(A)** annexin V-FITC stained cells in flow cytometry assay and **(B)** fluorescent cells in terminal deoxynucleotidyl transferase dUTP nick end label (TUNEL) assay. Scale bar = 100 μm.

Further evaluating the roles of JNK and PKCδ in PlGF-induced apoptosis, BEAS-2B and AEC II were pre-treated with JNK inhibitor or transfected with PKCδ siRNA to block the PlGF down-stream signaling pathways, then treated with 0–100 ng/ml PlGF for 24 h. Results of flow cytometry assay (Figure 4A) and TUNEL assay (Figure 4B) indicated that first, exogenous PlGF dose-dependently increased BEAS-2B and AEC II apoptotic levels and second, the JNK and PKCδ signaling pathways played crucial roles in PlGF-stimulated LE cell apoptosis.

The impact of NE-induced endogenous PlGF on NE-induced LE cell apoptosis was further evaluated in normal human bronchial epithelial cells (NHBE) with serum-free medium, which was the applicable condition for NE-digestion. This study also further proved that NE caused NHBE apoptosis and blocked endogenous PlGF signaling by VEGFR1 neutralized antibody, which attenuated the NE-induced NHBE apoptosis and NE-activated JNK and PKCδ signaling pathways (Additional file 1 and Additional file 4: Figure S3).

### Intra-tracheal instillation of NE increased PlGF expression and secretion and activated downstream JNK and PKCδ signaling pathways

**Figure 5:**
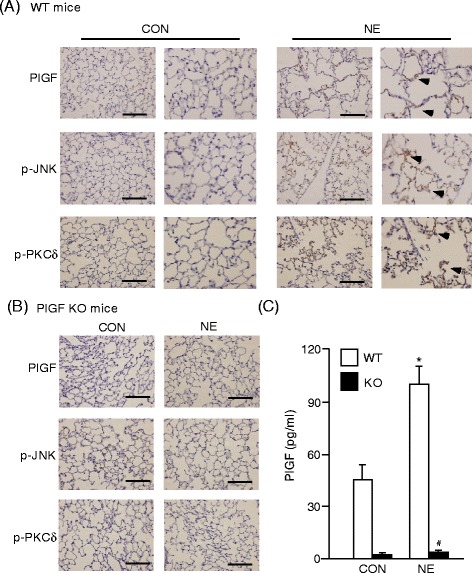
**NE increases expression of PlGF and activation of JNK and PKCδ signaling. (A and B)** Paraffin-embedded lung tissue sections were used for immuno-histochemistry (IHC) analysis and incubated with antibodies of PlGF, p-JNK and p-PKCδ. Arrow heads indicated positive stain of PlGF, p-JNK and p-PKCδ in LE cells. **(C)** Mice broncho-alveolar lavage fluid was analyzed for the PlGF level by ELISA. Scale bar = 200 μm. Data are presented as mean ± SEM. ^*^ *p* < 0.05 vs. vehicle-treated group; #*p* < 0.05 vs. corresponding WT group.

The role of PlGF in NE-induced LE cells apoptosis and emphysema was further confirmed in an animal model. Wild-type (C57BL/6) and PlGF KO mice were intra-tracheally treated with saline (CON) or 400 mU/ml NE (NE) weekly for one month. The pathology of the NE-treated mice showed elevated PlGF expression in alveolar epithelial cell (Figure 5A) and adjacent endothelial cells than controls (Additional file 2: Figure S1C). Moreover, NE-treated mice displayed more phosphorylated JNK and PKCδ levels than the control mice (Figure 5A).

In contrast, ablation of PlGF limited the expression of PlGF and blocked the NE instillation-induced activation of JNK and PKCδ (Figure 5B). The BAL fluid from NE-treated mice also had higher PlGF levels compared to the control mice. However, there was a lack of PlGF in KO mice (Figure 5C). These results demonstrated that NE instillation increased the expression and secretion of PlGF, as well as the activation of JNK and PKCδ in pulmonary cells.

### PlGF and PlGF-activated JNK and PKCδ pathways were involved in NE-induced apoptosis and emphysema in mice

**Figure 6:**
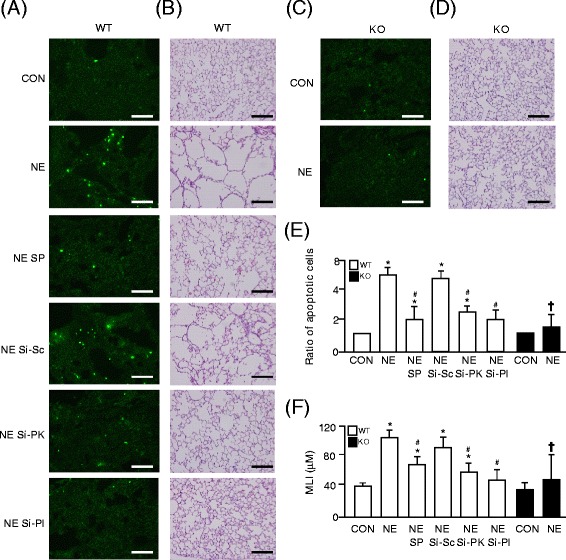
**NE-induced pulmonary apoptosis and emphysema are mediated by PlGF and the downstream JNK/PKCδ signaling pathways. (A and C)** The frozen lung tissue sections were used for TUNEL assay and **(B and D)** paraffin-embedded lung tissue sections were used for H and E staining. **(E)** Apoptotic cells in TUNEL assay were quantified and **(F)** the mean linear intercepts (MLI) from five independent sections were evaluated. Scale bar = 200 μm. Data are presented as mean ± SEM. ^*^ *p* < 0.05 vs. vehicle-treated group; #*p* < 0.05 vs. NE-treated group; †*p* < 0.05 vs. corresponding WT group.

To evaluate the roles of PlGF and JNK/PKCδ signaling in NE-induced apoptosis and emphysema in an animal model, 50 mg/kg of SP600125, 3 mg/kg scramble siRNA, 3 mg/kg PKCδ siRNA, or 3 mg/kg PlGF siRNA were co-treated with NE installation (NE SP, NE Si-Sc, NE Si-PK, or NE Si-Pl) on WT and PlGF KO mice weekly for one month. TUNEL assay indicated more abundant apoptotic cells in the pulmonary tissue of NE-treated mice than control mice (Figures 6A and E). In contrast, the ablation of PlGF protected mice from NE-induced pulmonary cell apoptosis (Figure 6C and E).

Moreover, NE-treated mice had the emphysema phenotype with enlargement of the alveolar space (Figure 6B), as evaluated by the mean linear intercept (MLI) (Figure 6F). On the other hand, ablation of PlGF protected mice from NE-induced pulmonary destruction (Figure 6D and F). Furthermore, blocking the JNK and PKCδ signaling pathways (NE SP and NE Si-PK) and silencing of PlGF (NE Si-Pl) abrogated the levels of NE-induced pulmonary apoptosis (Figure 6A and E) and attenuated the airspace enlargement in mice (Figure 6B and F). Thus, the animal model of elastase-instillation further confirmed that the NE-increased pulmonary PlGF and the PlGF-activated JNK/PKCδ signaling pathways were involved in NE-induced pulmonary apoptosis and emphysema *in vivo*.

## Discussion

There are several conserved trans-elements within the human and mouse PlGF promoter regions, including MRE and HRE [31,32]. Treatment with PlGF does not affect the expressions of MTF-1 and HIF-1α, which are the binding proteins for MRE and HRE. A conserved Egr-1 response element (CCCCGCCCC) [36] is observed near the transcriptional start site in both mouse and human PlGF promoter. Egr-1 is a rapid response transcription factor for UV and cigarette smoke stimuli that up-regulates several genes, including PTEN, microtubule-associated protein-1 light chain 3, and PAR-1 in LE cells [36-39]. The Egr-1-upregulated down-stream genes mediate various cellular functions like cell growth, proliferation, differentiation, and apoptosis [39]. Egr-1 also has an impact on the pathogenesis of acute lung injury [40]. A previous study has demonstrated that NE inhibitors decrease ventilator-induced Egr-1 expression [41]. In the present study, NE promotes the transient expression of Egr-1, which is involved in NE-induced PlGF expression.

The present study demonstrates that NE-induced PlGF promotes LE cell apoptosis, which corroborate the results of a previous study [22]. However, unlike previously established mechanisms of NE-induced LE cell apoptosis [19,20], this study is the first to show that NE-induces LE cell apoptosis through PlGF and PlGF-mediated downstream JNK and PKCδ signaling pathways. The results of NHBE cells further indicate that NE-promoted endogenous PlGF contributes to LE cell apoptosis. Furthermore, NE up-regulates PlGF in endothelial cells and in LE cells. The PlGF-induced LE cell apoptosis may work through both autocrine and paracrine mechanism. In addition, it is interesting to know that the up-regulation of PlGF is identified in an ovalbumin-induced asthma mice model wherein PlGF promotes neutrophilic chemotaxis [42]. Therefore, the positive feedback loop between NE and PlGF in the pathogenesis of COPD warrants further investigation.

Because of frequently ignored early symptoms and irreversible pulmonary damage, COPD remains a major cause of death worldwide [2]. As a chronic disease with insidious pathogenesis, COPD is difficult to diagnose early. Useful diagnostic markers will help in the early diagnosis, early treatment, and reduction of mortality and morbidity. A previous report indicates that the NE-digested product, Aα-Val360, may be a marker for COPD [43]. However, endogenous elastin fragments can disturb the utility of Aα-Val360 for predicting COPD.

The present study demonstrates that PlGF, which physiologically appears only in the embryonic stage, may be a suitable candidate as a diagnostic marker of early COPD. Based on the IHC results and BAL data in a previous study [26], COPD patients secrete and express more PlGF compared to non-COPD controls. Other than COPD, the up-regulation of PlGF is also associated with higher risk of several human diseases, including age-related macular degradation, sickle cell disease, and most kinds of tumors [24]. As PlGF expression is barely detectable in healthy adults, further investigation regarding the association between PlGF and COPD may therefore support PlGF as a candidate marker for early COPD.

A previous study indicates that mouse PlGF activates p38 MAPK and JNK signaling pathway in mouse alveolar epithelial cells, and that MLE-15 and human PlGF activates the p38 MAPK and JNK signaling pathway in BEAS-2B. In the present study, PlGF promotes only JNK and PKCδ in AEC II cell. The difference in cell systems may explain why PlGF acts through different down-stream signaling pathways. However, the JNK, p38 MAPK, and PKCδ signaling pathways should all be considered as potential therapeutic targets aside from PlGF for COPD therapy [44-46].

## Conclusions

Using human and mouse LE cells as well as an *in vivo* model, this study demonstrates that NE challenge stimulates PlGF expression and secretion, and that PlGF promotes LE cell apoptosis via the JNK and PKCδ signaling pathways. Thus, PlGF and the downstream JNK/PKCδ signaling pathways participate in the pathogenesis of CS-related COPD and should be considered potential therapeutic targets for COPD therapy.

## Additional files

Additional file 1:
**Supplemental materials and methods.**
Additional file 2: Figure S1. Neutrophil elastase (NE) increases placenta growth factor (PlGF) expression in endothelial cell. BAEC and fibroblast were treated with neutrophil elastase (NE) (0–300 mU/ml) for 24 h (A and B) and the cellular lysate were applied for Western blot analysis. (C) Wild type (WT) mice were intra-tracheally instilled with saline and 400 mU/ml NE weekly for one month. Paraffin-embedded lung tissue sections were used for immunohistochemistry (IHC) analysis and incubated with antibodies of PlGF. The arrow heads in enlarge figures indicated positive stain of PlGF only showed in endothelial cells of NE group. Data were presented as mean ± SEM. ^*^ *p* < 0.05 vs. vehicle-treated group. Additional file 3: Figure S2. PlGF-activated JNK and PKCdelta signaling pathways have no crosstalk in primary mouse alveolar type II epithelial cell (AEC II). (A and B) AEC II were transfected with PKCdelta siRNA for 24 h (A) or pretreated with SP600125 for 2 h (B) then treated with PlGF (100 ng/ml) for 0–24 hr. Cellular lysates were subjected to Western blot analysis with antibodies for phosphorylated JNK (p-JNK) and JNK (A); phosphorylated PKCδ (p-PKCδ) and PKCδ (B). Data were presented as mean ± SEM. ^*^ *P* <0.05 *vs.* vehicle-treated group. Additional file 4: Figure S3. NE-upregulated endogenous PlGF promotes apoptosis and activates JNK and PKCdelta signaling pathways in primary normal human bronchial epithelial (NHBE). (A and B) NHBE cells were treated with NE (300 mU/ml) for 0–60 h. Cellular lysates were subjected to caspase-3 activity (A) and trypanblue inclusion assay (B). NHBE cells were pretreated with FLT1 neutralizing antibody or IgG for 2 h then treated with NE (300 mU/ml) for 60 h. Cellular lysates were subjected to Caspase-3 activity (A) and trypanblue inclusion assay (B) and Western blot analysis with antibodies for phosphorylated JNK (p-JNK), phosphorylated PKCδ (p-PKCδ), JNK and PKCδ (C). Data were presented as mean ± SEM. ^*^ *P* <0.05 *vs.* vehicle-treated group. ^#^ *P* <0.05 *vs.* PGF-treated group.

## Abbreviations

COPD: Chronic pulmonary obstructive disease
CS: Cigarette smoke
NE: Neutrophil elastase
MMP: Matrix metalloprotease
LE: Lung epithelial
PlGF: Placenta growth factor
AEC II: Type II alveolar epithelial cell
MAPK: Mitogen-activated protein kinase
BAL: Broncho-alveolar lavage
WT: Wild-type
Egr-1: Early growth response gene-1
JNK: c-Jun N-terminal kinase
PKC: Protein kinase C
MTF: Mental-regulatory transcription factor
HIF: Hypoxia inducible factor
ELISA: Enzyme-linked immuno-sorbent assay
ChIP: Chromatin immuno-precipitation
NHBE: Normal human bronchial epithelial cells
KO: Knockout
TUNEL: Terminal deoxynucleotidyl transferase dUTP nick end labeling
IHC: Immuno-histochemistry
MRE: Metal response element
HRE: Hypoxia response element
PAR: Protease-activated receptor
