# Supplementary material for: PlGF mediates neutrophil elastase-induced airway epithelial cell apoptosis and emphysema
Source: Respir Res. 2014 Sep 5;15(1):106. doi: 10.1186/s12931-014-0106-1 (PMC4267747; doi:10.1186/s12931-014-0106-1)
Supplement: Additional file 1: — Supplemental materials and methods. [file 12931_2014_106_MOESM1_ESM.doc]

**Supplemental Materials**

H**uman** FLT1 neutralizing antibody (AF321) and IgG were **obtained from** R&D Systems. Bovine aortic endothelial cell (BAEC) (B304-05) was purchase from Cell Application Inc. and human skin fibroblast (ATCC CRL-1508) was purchase from ATCC. Primary normal human bronchial epithelial (NHBE) cells were kindly provided by Dr. Reen Wu at University of California, Davis.

**Supplemental Methods**

**Trypan blue exclusion assay.** Treated- or untreated-NHBE cells were treated with NE (300mU/ml) for 0-60 h and then stained with trypan blue (1:1 in volume, Sigma, St. Louis, MO, USA, T6164), and the cells that were not stained blue were counted using a hemacytometer counting chamber (Sigma, Z359629 ).

**CASP3 activation assay.** Treated- or untreated- NHBE cells were analyzed for CASP3 activity by using an assay kit (Merck-Millipore, QIA70) according to the manufacturer’s instructions with Packard LumiCount (Molecular Devices, Sunnyvale, CA, USA, BL10000).
