# Supplementary figures and images for: PlGF mediates neutrophil elastase-induced airway epithelial cell apoptosis and emphysema
Source: Respir Res. 2014 Sep 5;15(1):106. doi: 10.1186/s12931-014-0106-1 (PMC4267747; doi:10.1186/s12931-014-0106-1)

Fig. S1

(A) Endothelial cell

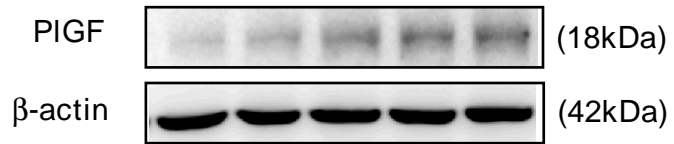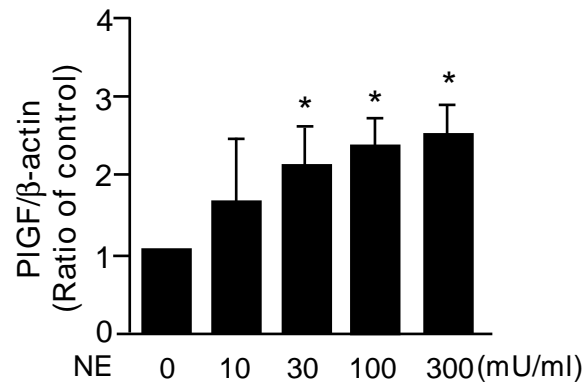

(B) Fibroblast

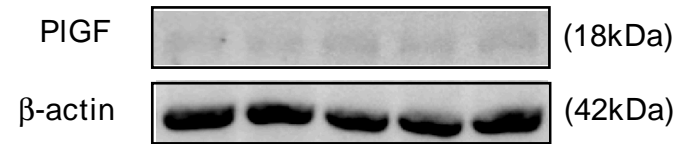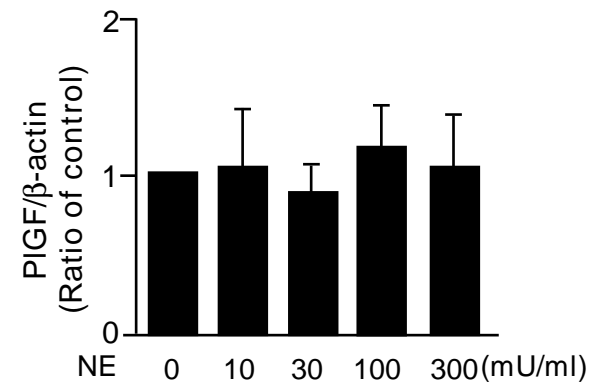

(C)

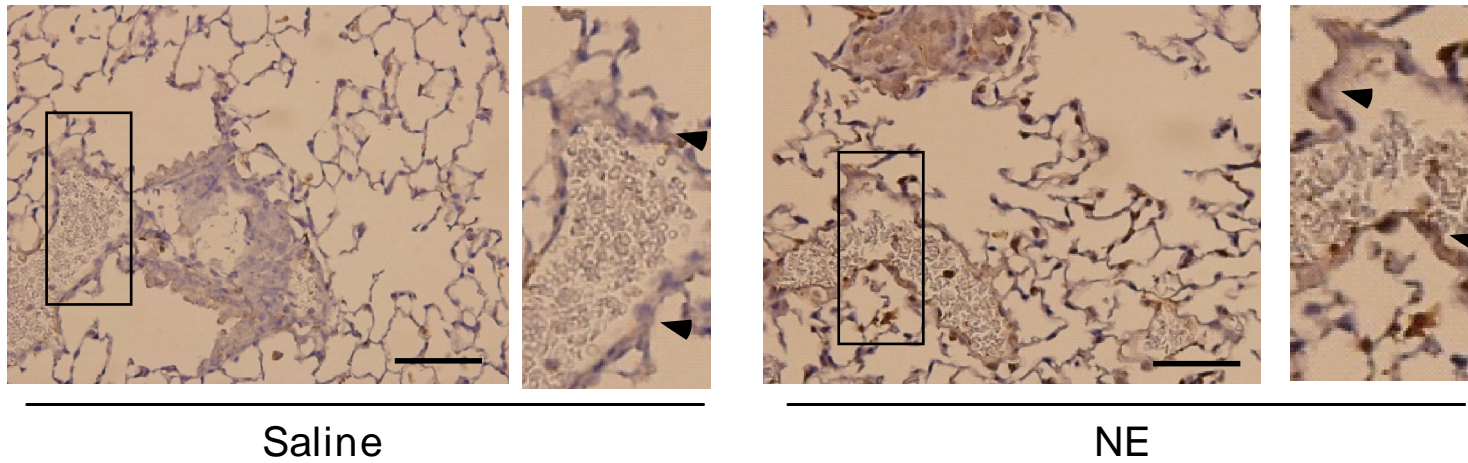

Supplement: Additional file 2: Figure S1. — Neutrophil elastase (NE) increases placenta growth factor (PlGF) expression in endothelial cell. BAEC and fibroblast were treated with neutrophil elastase (NE) (0–300 mU/ml) for 24 h (A and B) and the cellular lysate were applied for Western blot analysis. (C) Wild type (WT) mice were intra-tracheally instilled with saline and 400 mU/ml NE weekly for one month. Paraffin-embedded lung tissue sections were used for immunohistochemistry (IHC) analysis and incubated with antibodies of PlGF. The arrow heads in enlarge figures indicated positive stain of PlGF only showed in endothelial cells of NE group. Data were presented as mean ± SEM. * p < 0.05 vs. vehicle-treated group. [file 12931_2014_106_MOESM2_ESM.pdf]

Fig. S2

(A)

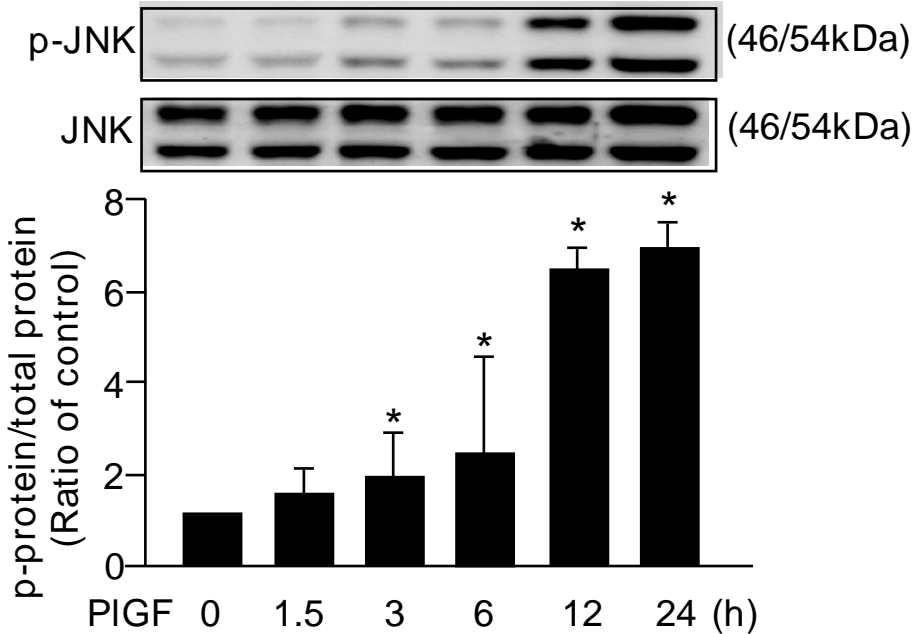

(B)

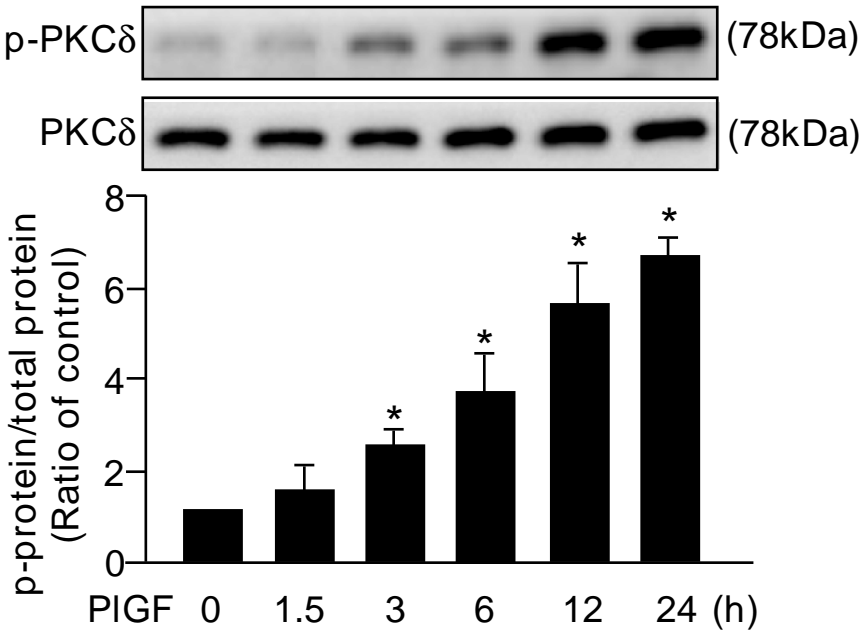

Supplement: Additional file 3: Figure S2. — PlGF-activated JNK and PKCdelta signaling pathways have no crosstalk in primary mouse alveolar type II epithelial cell (AEC II). (A and B) AEC II were transfected with PKCdelta siRNA for 24 h (A) or pretreated with SP600125 for 2 h (B) then treated with PlGF (100 ng/ml) for 0–24 hr. Cellular lysates were subjected to Western blot analysis with antibodies for phosphorylated JNK (p-JNK) and JNK (A); phosphorylated PKCδ (p-PKCδ) and PKCδ (B). Data were presented as mean ± SEM. * P <0.05 vs. vehicle-treated group. [file 12931_2014_106_MOESM3_ESM.pdf]

Fig. S3

(A)

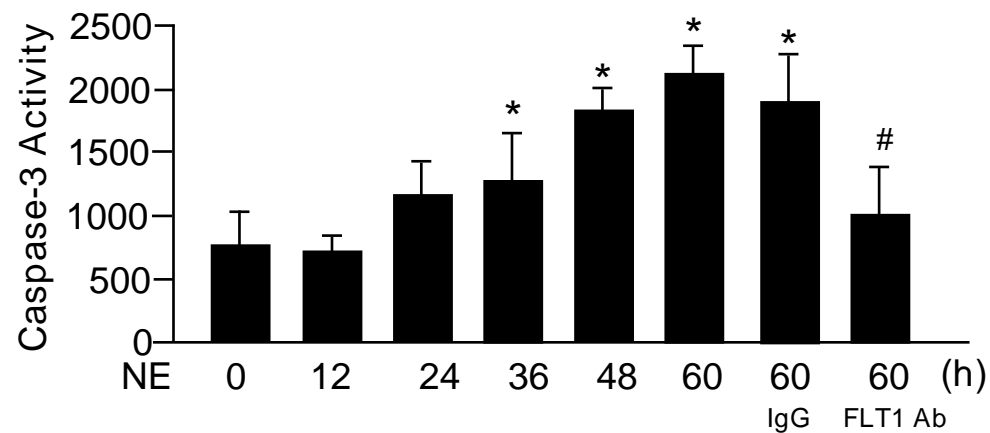

(B)

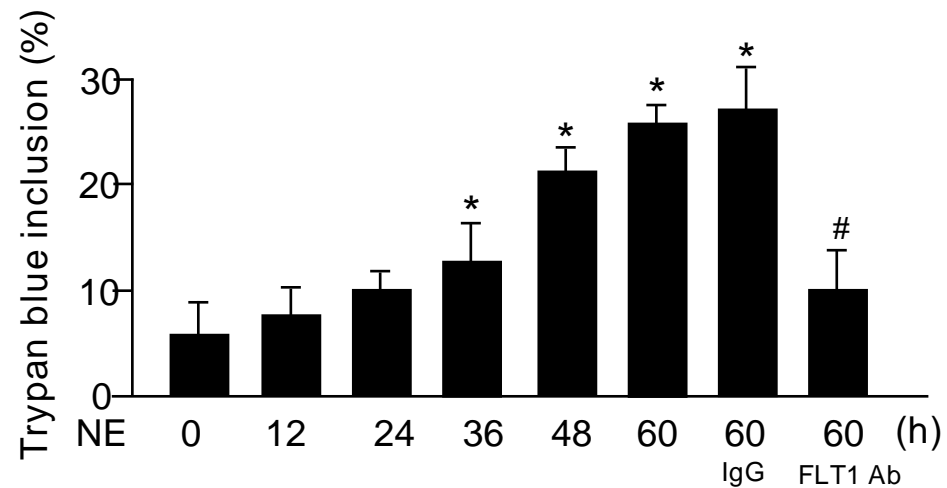

(C)

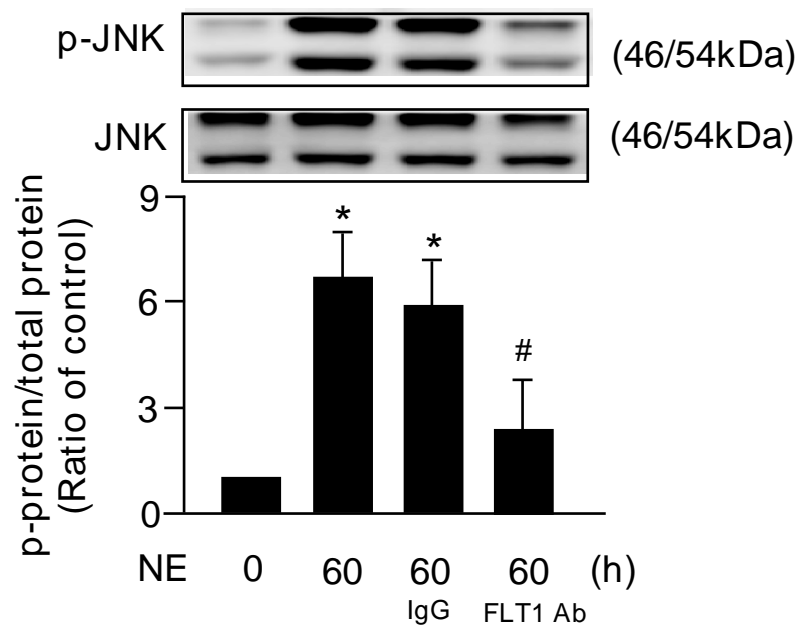

(D)

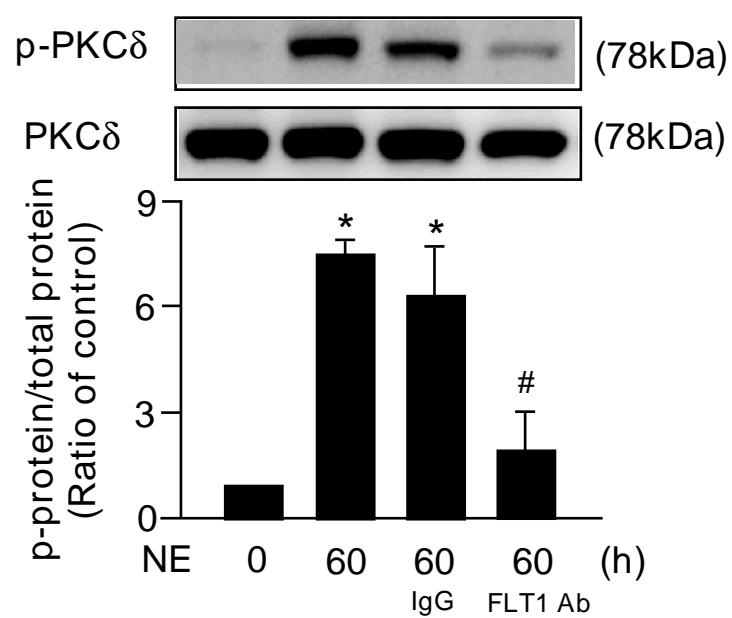

Supplement: Additional file 4: Figure S3. — NE-upregulated endogenous PlGF promotes apoptosis and activates JNK and PKCdelta signaling pathways in primary normal human bronchial epithelial (NHBE). (A and B) NHBE cells were treated with NE (300 mU/ml) for 0–60 h. Cellular lysates were subjected to caspase-3 activity (A) and trypanblue inclusion assay (B). NHBE cells were pretreated with FLT1 neutralizing antibody or IgG for 2 h then treated with NE (300 mU/ml) for 60 h. Cellular lysates were subjected to Caspase-3 activity (A) and trypanblue inclusion assay (B) and Western blot analysis with antibodies for phosphorylated JNK (p-JNK), phosphorylated PKCδ (p-PKCδ), JNK and PKCδ (C). Data were presented as mean ± SEM. * P <0.05 vs. vehicle-treated group. # P <0.05 vs. PGF-treated group. [file 12931_2014_106_MOESM4_ESM.pdf]
